# Anticancer Activity and Apoptosis Induction of Gold(III) Complexes Containing 2,2′-Bipyridine-3,3′-dicarboxylic Acid and Dithiocarbamates

**DOI:** 10.3390/molecules26133973

**Published:** 2021-06-29

**Authors:** Ali Alhoshani, Adam A. A. Sulaiman, Homood M. As Sobeai, Wajhul Qamar, Moureq Alotaibi, Khalid Alhazzani, Muhammad Monim-ul-Mehboob, Saeed Ahmad, Anvarhusein A. Isab

**Affiliations:** 1 Department of Pharmacology and Toxicology, College of Pharmacy, King Saud University, P.O. Box 2457, Riyadh 11451, Saudi Arabia; hassobeai@KSU.EDU.SA (H.M.A.S.); wqidris@KSU.EDU.SA (W.Q.); mralotaibi@KSU.EDU.SA (M.A.); Kalhazzani@ksu.edu.sa (K.A.); 2 Department of Chemistry, King Fahd University of Petroleum and Minerals, Dhahran 31261, Saudi Arabia; adamahmed@kfupm.edu.sa; 3 Department of Chemistry, Government College of Science, Wahdat Road, Lahore, Pakistan; mmmehboob786@hotmail.com; 4 Department of Chemistry, College of Sciences and Humanities, Prince Sattam bin Abdulaziz University, Al-Kharj 11942, Saudi Arabia; saeed_a786@hotmail.com

**Keywords:** gold(III), 2,2′-bipyridine-3,3′-dicarboxylic acid, anti-cancer activity, dithiocarbamates, apoptosis

## Abstract

Three novel gold(III) complexes (**1**–**3**) of general composition [Au(Bipydc)(S_2_CNR_2_)]Cl_2_ (Bipydc = 2,2′-bipyridine-3,3′-dicarboxylic acid and R = methyl for dimethyldithiocarbamate (DMDTC), ethyl for diethyldithiocarbamate (DEDTC), and benzyl for dibenzyldithiocarbamate (DBDTC)) have been synthesized and characterized by elemental analysis, FTIR and NMR spectroscopic techniques. The spectral results confirmed the presence of both the Bipydc and dithiocarbamate ligands in the complexes. The in vitro cytotoxic studies demonstrated that compounds **1**–**3** were highly cytotoxic to A549, HeLa, MDA-231, and MCF-7 cancer cells with activities much higher (about 25-fold) than cisplatin. In order to know the possible mode of cell death complex **2**, [Au(Bipydc)(DEDTC)]Cl_2_ was further tested for induction of apoptosis towards the MCF-7 cells. The results indicated that complex **2** induces cell death through apoptosis.

## 1. Introduction

The clinical success of platinum anti-cancer drugs [1,2,3,4] prompted a great deal of attention towards the development of isoelectronic gold(III) anti-cancer complexes with the anticipation that they might have activity similar to that of platinum(II) anti-tumor drugs [5,6,7,8,9,10,11,12,13,14,15,16,17]. Both gold(III) and platinum(II) give rise to square planar geometries; but the high redox potential for gold(III) compounds, their relatively poor stability, and faster ligand substitution reactions could impede their application as medicinal agents under normal physiological conditions [5,6,7,8,9,10,11,12,13,14,17,18]. However, the coordination of bidentate and polydentate ligands, such as 2,2′-bipyridine and terpyridine, provides sufficient stabilization to gold(III) compounds under physiological conditions and shows favorable anti-tumor properties [7,8,11,12,13,14,15,16].

As a result, assorted sets of a potential anti-tumor gold(III) compounds were synthesized and characterized, which exhibited significant anti-proliferative effects in vitro under similar physiological conditions. They include the gold(III) complexes of bipyridines [11,12,13,19,20,21,22,23], diamines [16,17,23,24,25,26,27,28,29], quinolones [16], dithiocarbamates [30,31,32,33,34,35,36,37], and porphyrinates [38,39], as well as dinuclear gold(III) complexes [13,22,23] and a variety of organogold(III) compounds [37,40,41,42].

Mechanistic studies on the cytotoxic gold(III) complexes indicate that these complexes show a weak binding affinity to DNA, suggesting that they exert anti-proliferative effects, mainly through DNA-independent mechanisms [7,11,12,13,43]. The compounds of gold(III) have shown high reactivity to various protein models indicating that the interactions between gold and proteins are responsible for their cytotoxic effects [7,13,23,33,44,45,46,47]. The apoptosis for such compounds can be caused by direct mitochondrial damage [42,48]. The key targets for these gold compounds are some unusual proteins, for example mitochondrial selenoprotein, thioredoxin reductase, the proteasome and the nuclear factor kb (NF-kb) [32,33,47,48,49,50]. The strong inhibition of thioredoxin reductase could ultimately lead to the profound change of the mitochondrial membrane potential, to cause cytochrome *c* release and subsequent excitation of apoptosis [49].

Gold(III) complexes containing bipyridine ligands have been extensively investigated for the cytotoxic properties by Messori and other groups [11,12,13,22,23]. These complexes exist in mononuclear [13] as well as dinuclear forms [22]. The gold(III) atom in [Au(bipy)X_2_] + complexes has a square-planar geometry, attained by two N atoms from the bipyridine and two halide/pseudohalide ions. Angela Casini and co-workers have reported the synthesis and anti-cancer properties of the gold(III) series of compounds containing bipyridine derivatives. They noted that the compound with methylated bipyridine showed more cytotoxic activity against A2780 (the human ovarian carcinoma cell line) in comparison with methoxylated, aminated, and even no-branched derivatives [13]. Amani et al. elaborated on the study by using different anions for the cationic gold(III) bipyridine derivative [21]. Counter anions have been shown to play a major role in the cytotoxicity of the related compounds in various cell lines. The complexes with the relevant anionic part of [AuX_4_]^−^ (X = Cl and Br) displayed good agreement in their anti-cancer activities toward human cancer cell lines.

**Scheme 1:**
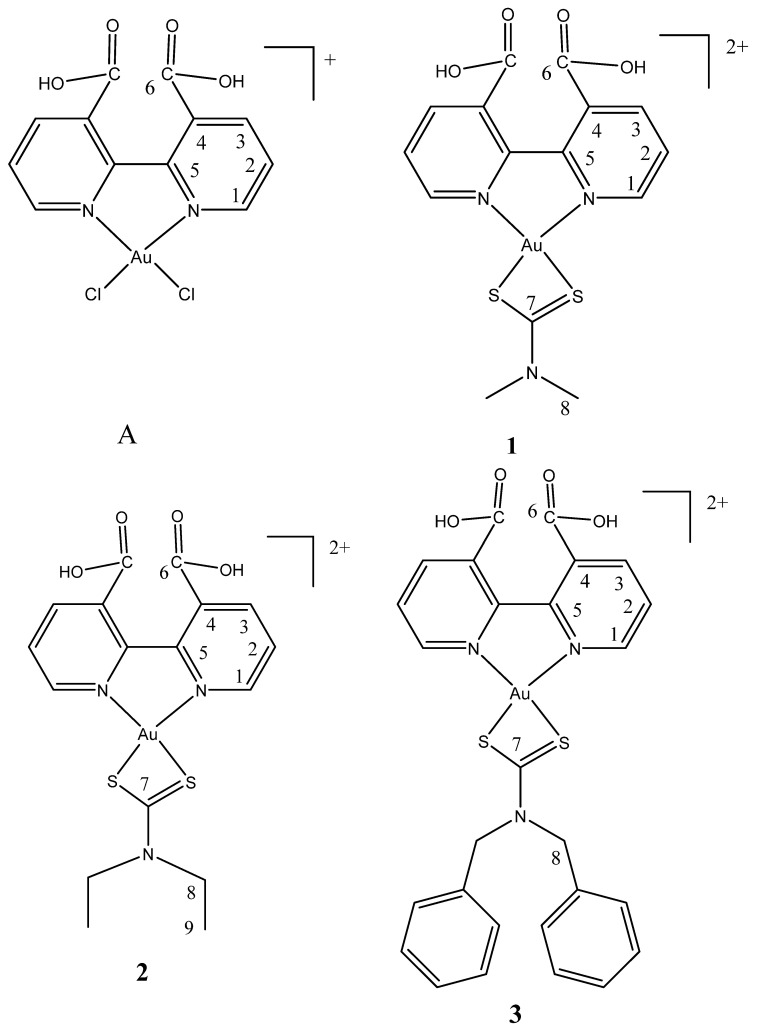
The possible structures of gold(III) complexes along with resonance assignment. In all cases, counter ions are chloride.

Due to their strong cell growth-inhibitory effects, gold(III)-dithiocarbamates have received significant attention as potential drugs for cancer treatment [30,31,32,33,34,35,36,37]. In this context, we have taken advantage of the idea of linking the well-known gold(III)-based bipyridine moiety with the effective chemo-protective function of dithiocarbamates, which proved to be an efficient strategy [19,20]. In an effort to develop more effective anti-cancer agents with reduced toxicity, we report here the synthesis, spectral characterization, in vitro anti-tumor activities, DNA damage, and cell death studies of three new gold(III) compounds (**1**–**3**) using 2,2′-bipyeidine-3,3′-dicarboxylic acid ligand (Bipydc) along with three dithiocarbamates. The possible structures of the investigated complexes are shown in Scheme 1.

## 2. Results and Discussion

### 2.1. Synthesis and Spectroscopic Characterization

The complexes were prepared by treating Na[AuCl_4_]·2H_2_O, 2,2′-bipyridine-3,3′-dicarboxylic acid, and sodium salts of dithiocarbamates in 1:1:1 stoichiometric ratio in ethanol. The complexes were isolated as dry powder solids, soluble in DMSO and chloroform. The stoichiometry of complexes **1**–**3** was formulated on the basis of elemental analysis. The complexes **1**–**3** are assumed to be a classic mononuclear species with square-planar geometry [31,32,33,37]. In all cases the gold(III) center is stabilized by the presence of two nitrogen ligands, and coordination of dithiocarbamates takes place through the sulfur-donating atoms of the NCSS moiety in a bidentate symmetrical mode.

Due to the steric effects of the substituents placed at the 3,3′-positions, the two pyridine rings of the bipyridyl ligand (Bipydc) in the complexes are not coplanar [51]. The structure of the platinum(II) complex [Pt(Bipydc)Cl_2_]·2DMF shows that Bipydc is coordinated to platinum through its nitrogen atoms. However, the coordinated pyridine rings are twisted by 26.5°, relative to one another, owing to the steric repulsion between the carboxylic acid groups in the 3,3′-positions of Bipydc [52]. A similar structural arrangement is predicted for the present series of the complexes. In some cases, coordination through carboxyl groups is also observed. For example, in a recent report on copper(II) coordination polymer with 2,2′-bipyridyl-3,3′-dicarboxylic acid {[Cu(Bipydc)(H_2_O)]·2H_2_O}_n_, the ligand is found to coordinate through both the nitrogen and oxygen atoms of the carboxyl group, but to different metal ions [53].

The selected IR frequencies of the ligands and their gold(III) complexes are given in Table 1. In the IR spectra of the complexes (**1**–**3**), a strong band around 1500 cm^−1^ is associated with the C-N stretching vibration of the N–CSS^−^ moiety of dithiocarbamates [30,31,32,37,54]. This value defines a carbon–nitrogen bond order intermediate between a single bond (ν = 1350–1250 cm^−1^) and a double bond (ν = 1690–1640 cm^−1^) [54]. The C = N stretching mode of the bipyridine ligand also falls in the same region [13,21] and is therefore indistinguishable from this band. The higher-frequency shifts in ν(N–CSS) mode of dithiocarbamates upon coordination are consistent with an increase in the C–N double bond character and support the bidentate coordination of the S atoms of (-CSS) moieties to the metal center. The bands in the region of the 1100–900 cm^–1^ represent the symmetrical and asymmetrical vibrational modes of (CSS) and (CSS) stretching, respectively. The C–H (aromatic) and C–H (aliphatic) stretching vibrations appear around 3000 and 2900 cm^−1^, respectively. In addition, the signal at about 3400 cm^−1^ represents the O-H absorptions of the COOH groups of Bipydc ligands. The appearance of this band indicates that the carboxylate ions are not involved in complexation. The presence of the absorption band characteristics of Bipydc and dithiocarbamates suggests the coordination of both ligands to the gold(III) center.

The ^1^H and ^13^C-NMR chemical shifts of the complexes are provided in Table 2 and Table 3, respectively. In the ^1^H NMR spectra of complexes **1** and **2**, the N(CH) protons of dithiocarbamates were detected between 3 and 4 ppm and around 5 ppm for **3**. The splitting pattern of the protons attached to the R groups is usual. The ^1^H NMR spectrum of complex **3** shows multiple peaks centered between δ 7.22 and 7.33 ppm, due to the protons of the benzene ring. The aromatic protons of bipyridine resonate around 7 ppm. The N = C-H proton appeared at the most downfield position of about 8.8 ppm.

The ^13^C{^1^H} NMR spectra of the complexes exhibit signals due to the N–CH and CS2 carbons of dithiocarbamates near 50 and 200 ppm, respectively. A significant upfield shift in the CS2 resonance is attributed to the lowering of the C = S bond order upon coordination and a shift of N-C electron density, producing a partial double bond character in the C–N bond. The aromatic carbons of the bipyridine ring resonate between 120 and 160 ppm. Downfield shifts in C = N resonance of the ligands upon complexation indicates the binding of Bipydc to gold(III) through the imine nitrogen atom. The peaks due to carboxylate carbons are observed near 167 ppm. The ^1^H and ^13^C-NMR chemical shift values are in accordance with the literature reports [13,20,21,30,54].

### 2.2. Cytotoxic Activity

**Figure 1:**
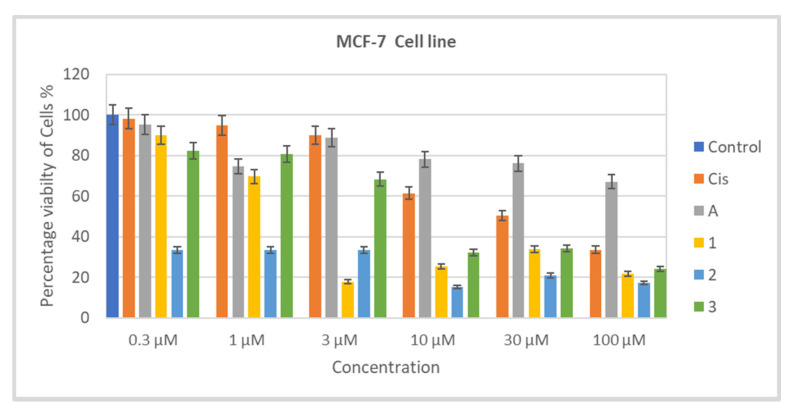
Effect of the concentration of complexes **A**, **1**–**3** and cisplatin on the viability of MCF-7 cells.

**Figure 2:**
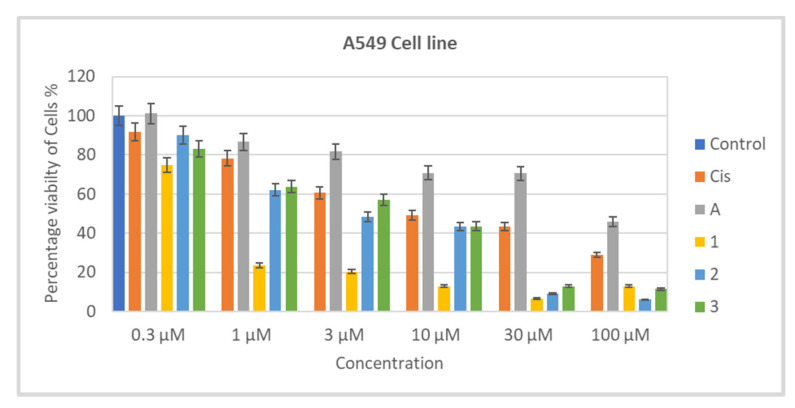
Effect of the concentration of complexes **A**, **1**–**3** and cisplatin on the viability of A549 cells.

**Figure 3:**
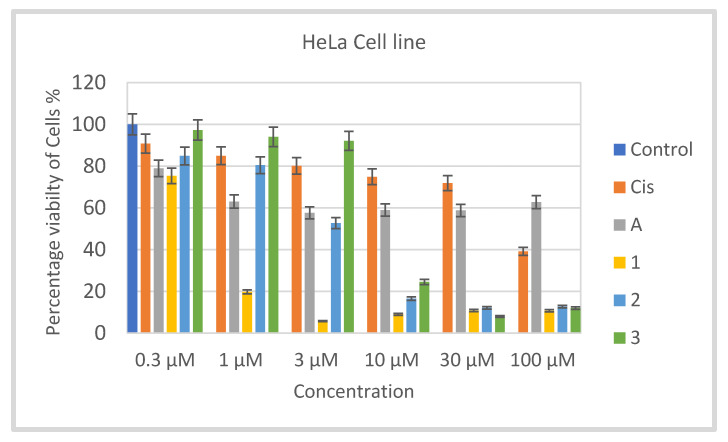
Effect of the concentration of complexes **A**, **1**–**3** and cisplatin on the viability of HeLa cells.

**Figure 4:**
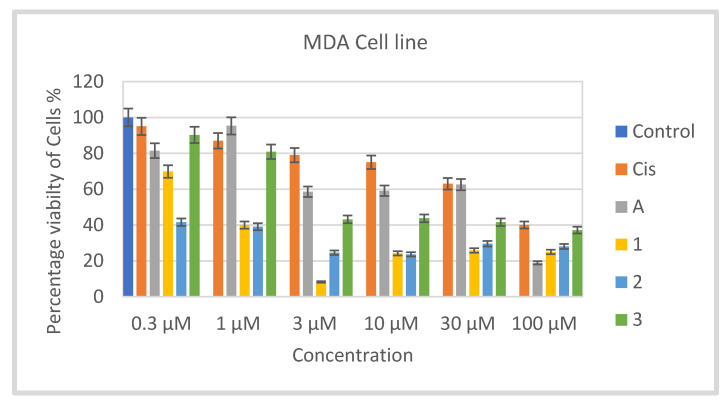
Effect of the concentration of complexes **A**, **1**–**3** and cisplatin on the viability of MDA cells.

The in vitro cytotoxicity of synthesized gold(III) complexes (**A**, **1**–**3**) was evaluated via MTT assays towards four cell lines: A549, HeLa, MDA-231, and MCF-7. The MTT signal is mainly influenced by the cellular metabolic activity, where higher metabolic activity of the cells will produce higher MTT signals, and vice versa. For comparison, the anti-cancer activity of cisplatin was also examined under the same conditions. The corresponding IC_50_ values are given in Table 4. Comparisons of cell viability percentages for all cell lines are shown in Figure 1, Figure 2, Figure 3 and Figure 4. All four complexes (A,**1**–**3**) displayed excellent cytotoxic activity, as compared to cisplatin. In most cases, the IC_50_ value of the complexes (**1**–**3**) was about 3 µM, while for cisplatin it was around 9 µM (except for MCF-7, 31 µM). The cytotoxic effects of the complexes are comparable to each other against the selected four cells. It has been observed that the activity is enhanced upon the complexation of dithiocarbamates. Complex **1** was found to be the most effective against HeLa cells, **2** against MCF-7, and **3** against A549 cells. Figure 4 showed that complex **1** unexpectedly produced higher MTT signals at higher concentrations (10, 30, and 100 µM) compared to lower concentrations (3 µM) in MDA-231 cells. A possible explanation for the disparity of MTT signals between higher and lower concentrations is that at higher concentration complex **1** might have induced oxidative burst, which subsequently increased the metabolic activity of MDA cells, leading to the magnification of MTT signals despite the overall cellular death. The higher cytotoxicity of the complexes may be related to the strong binding of dithiocarbamate ligands with the gold(III) ion, which would increase its stabilization. Since cancer cells have a higher proliferative capacity than normal cells, the complexes are expected to target tumor cells via the induction of reactive oxygen species leading to DNA damage and cellular death [55,56]. The comparable cytotoxicity of the complexes reflects that the structure of dithiocarbamate has little influence on the anti-tumor properties of the complexes. Although the anti-tumor activities of complexes **1**–**3** are significant, they seem poor compared to those reported earlier for such complexes [20]. Further in vivo studies using mice models should be carried out to evaluate the safety of major organs and the efficacy of anti-cancer properties. Since these complexes bear structural similarities to cisplatin, it is proposed to use cisplatin as a positive control for dosing and comparison.

### 2.3. Apoptosis Induction

**Figure 5:**
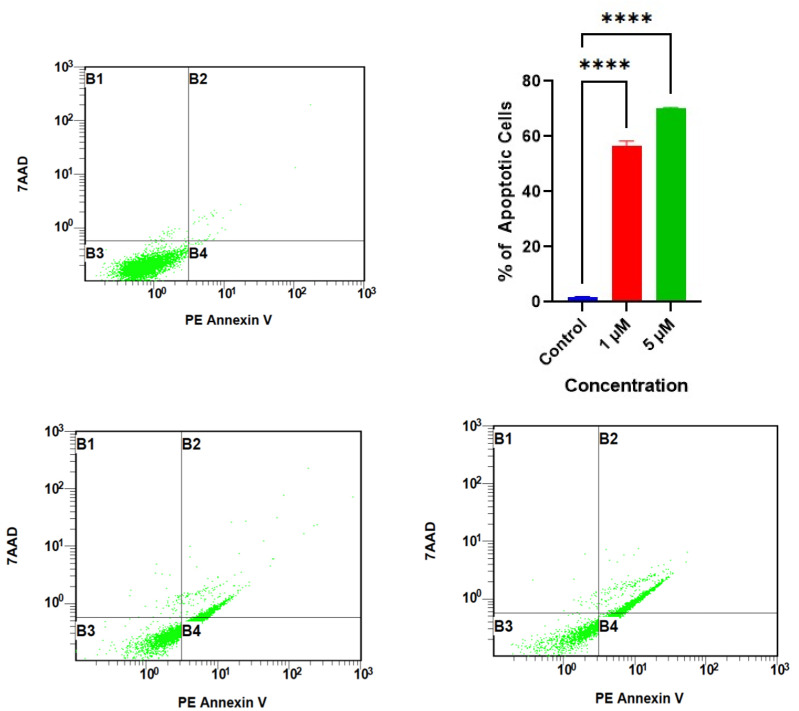
Apoptotic evaluation by Annexin V staining of MCF-7 cells treated with **2**. The (****) is significant compare to control.

To further investigate how the complexes inhibited proliferation, the induction of apoptosis for complex **2** was evaluated in MCF-7 cells after 24 h. The control, 1 μM, and 5 μM concentrations of the complex were used. The observed apoptotic effect was compared to the control and expressed in Figure 5. The results showed that complex **2** inhibited apoptosis proliferation. Distinct morphological characteristics, including cytoplasmic and nuclear condensation, breakdown in DNA, mucosal dysfunction, and microvilli, are usually associated with apoptosis. The exposure of phosphatidylserine (PS) is usually before plasma membrane integrity loss during apoptosis. Healthy cells do not react to annexin V, a marker of PS, while apoptosis-prone cells are reactive to annexin V [57]. Figure 5 shows the percentage of apoptotic cells (MCF-7) increased in a dose-dependent manner. The total percentage rates of early apoptosis and late apoptosis for MCF7 were 1.68 ± 0.33%, 56.78 ± 1.50%, and 70.07 ± 0.42%, respectively. It is clear that complex **2** induced apoptosis at a sub-toxic dose.

### 2.4. DNA Damage

**Figure 6:**
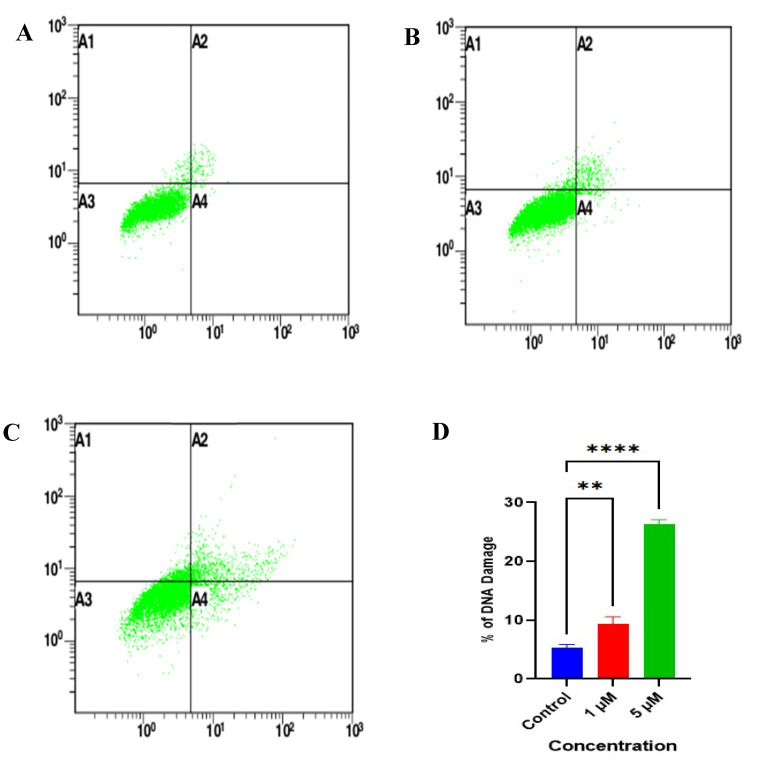
DNA damage-inducing effect of complex 2. In (**A**–**C**), A1: percentage of ATM-activated cells, A2: percentage of DNA double-strand breaks (dual activation of both ATM and H2A.X), A3: percentage of negative cells (e.g., no DNA damage), (**D**) the total DNA damage (A1 + A2 + A4). A significant increase in DNA strand breaks was observed in complex 2 treated cells in a dose-dependent manner. The (**) and (****) is significant compare to control.

Conventional anti-cancer agents are genotoxic. They induce DNA damage, crumble the cancer cell’s ability to proliferate, and force cells to undergo apoptosis. To assess whether complex **2** behaved in a manner similar to classic genotoxic chemotherapy drugs and that DNA damage was the underlying mechanism for the elevation of apoptosis, the total DNA damage of MCF7 cells was measured 24 h after treatment. Two treatment concentrations of complex solutions 1 μM and 5 μM were compared to control cells that were left with no treatment. The assay revealed significantly increased chromosomal DNA strand breaks upon addition of the complex in dose-dependent manner. The total DNA damage percentages of 1μM and 5μM treated apoptotic cells were 8% and 27%, respectively, compared to the control cells, which was 5% (Figure 6). These data suggest that complex **2** induced DNA insults in the treated cells leading to promote apoptosis.

### 2.5. Stability of Complexes ***1*** and ***3***

**Figure 7:**
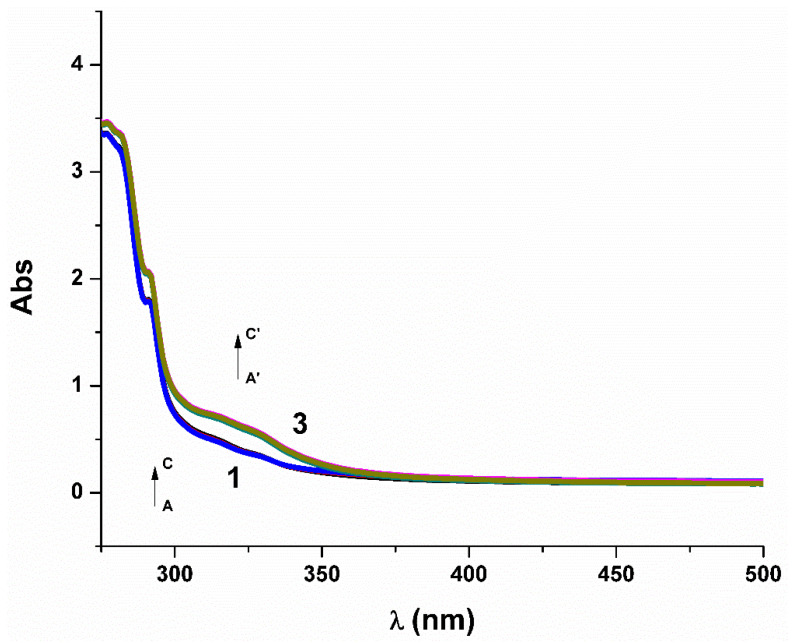
UV–vis spectra of 1 ppm complexes **1** and **3** taken in acetonitrile with the presence of 0.5% *v*/*v* BPS (pH 7.0) at room temperature.

The stability of complexes **1** and **3** was checked in acetonitrile with 0.5% *v*/*v* PBS (pH 7.0) at room temperature using UV–visible spectroscopy. The complexes are completely soluble in both acetonitrile and phosphate buffer solution (PBS, pH 7.0). The spectra were taken in the beginning and after 24 h and 72 h, as shown in Figure 7. No changes were observed in the spectra, indicating their stability in solution.

### 2.6. Effect on ROS Level

**Figure 8:**
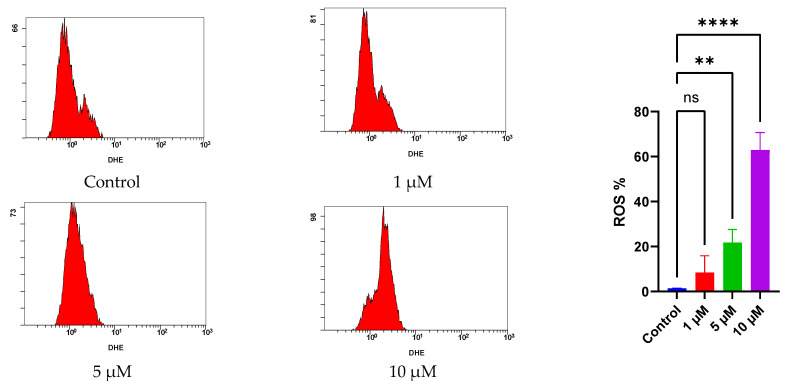
The effect of complex **2** concentrations on ROS levels in MCF-7 cells. The shift of the peaks toward the right shows increasing ROS accumulation in treated cells. The (**) and (****) is significant compare to control.

Flow cytometry analysis of ROS level was measured by using a fluorescent dihydroethidium (DHE) probe. Complex **2** treated MCF-7 cells indicated the progressive increase in ROS in a dose-dependent manner (1, 5, and 10 μM) as shown in Figure 8. The shift in the peaks towards the right as compared to the control indicates the increment in ROS levels in complex **2** treated MCF-7 cells. The accumulation of ROS is known to induce apoptosis, as confirmed by the results of a recent study [58].

### 2.7. Effect on Mitochondrial Membrane Potential

**Figure 9:**
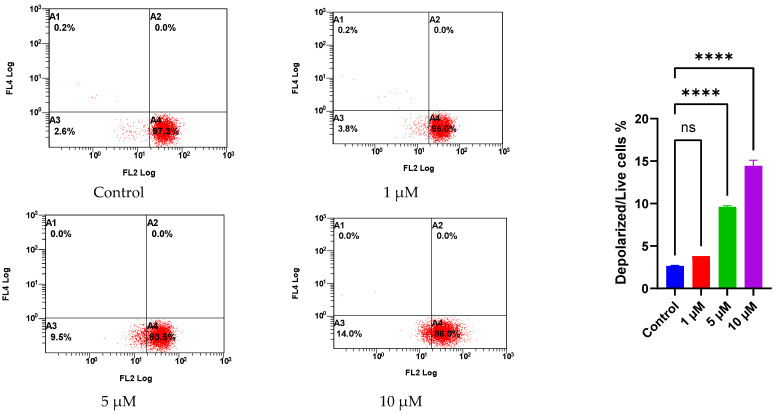
The effect of complex **2** concentrations on mitochondrial membrane potential in MCF-7 cells. The (****) is significant compare to control.

Apoptosis increases mitochondrial membrane permeability, lowering the transmembrane potential of the mitochondrial membrane. The decreasing mitochondrial membrane potential has been associated with apoptosis and DNA damage induction, indicating that apoptosis is also associated with the mitochondrial pathway**.** Figure 9 shows how the concentration of complex **2** (1, 5, and 10 μM) decreases the mitochondrial membrane potential. In the control group, membrane potential decrease was observed at 97.3% in MCF-7 cells, while the highest mitochondrial membrane potential decrease was observed at 86.0% with 10 μM complex **2**. The percentage indicates that the mitochondrial membrane potential decreases with increasing complex **2** concentration (1, 5, and 10 μM), indicating mitochondrial depolarization and apoptosis induction in complex **2** treated MCF-7 cells. This manner is consistent with the previous results that showed the increased apoptosis and DNA damage with the decreasing mitochondrial membrane potential in MCF-7 cells at a sub-toxic dose of complex **2** [58].

## 3. Experiment and Instrumentation

### 3.1. Materials

Sodium tetrachloridoaurate(III) dihydrate (NaAuCl_4_·2H_2_O), 2,2′-bipyridine-3,3′-dicarboxylic acid, and sodium salts of dimethyldithiocarbamate dihydrate, diethyldithiocarbamate, and dibenzyldithiocarbamate were purchased from Sigma Aldrich, Co. (St. Louis, MO, USA). Ethanol, diethyl ether, and dichloromethane were obtained from Fluka AG (St. Gallen, Switzerland). All solvents were of analytical grade and were used without further purification.

### 3.2. Instrumentation

The instruments used to characterize the prepared complexes have been reported in the literature [54].

### 3.3. Synthesis of [Au(Bipydc))Cl_2_]Cl (***A***)

It was prepared by combining 0.122 g (0.5 mmol) 2,2′-bipyridine-3,3′-dicarboxylic acid in 10 mL of ethanol and 0.20 g (0.5 mmol) NaAuCl_4_·2H_2_O in 10 mL of ethanol. The mixture was stirred for 3 h at room temperature and then filtered. The yellow product was washed twice with ethanol (5 mL) and three times with diethyl ether (10 mL), then dried in the dark and stored in a fridge. Yield = 0.212 g, 77.6%. Analysis (C_12_H_8_AuCl_3_N_2_O_4_, 547.52): Calcd. C 26.32, H 1.47, N 5.11; found C 25.98, H 1.53, N 5.54.

### 3.4. Synthesis of Complexes ***1***–***3***

In the first step, 0.122 g (0.5 mmol) 2,2′-bipyridine-3,3′-dicarboxylic acid in 10 mL of ethanol was added to 0.200 g (0.5 mmol) Na[AuCl_4_]·2H_2_O in 10 mL of ethanol, and the mixture was stirred for 3 h, generating a yellow product. In the second step, 0.5 mmol of the respective sodium dithiocarbamate in 5 mL of ethanol was added dropwise to the above mixture. The mixture was stirred for an additional 10 min. For complexes **1** and **2**, yellow precipitates were obtained, while in the case of **3**, it was an orange color. The products were collected by filtration, washed with distilled water (3 × 10 mL), and dried under vacuum. Yield: 0.215 g, 68% for **1**; 0.245 g, 74.2% for **2**; 0.278 g, 71% for **3**.

### 3.5. Analysis

(C_15_H_14_AuCl_2_N_3_O_4_S_2_, 632.30): Calcd. C 28.49, H 2.23, N 6.64, S 10.14; found C 28.21, H 2.36, N 6.54, S 9.98.

(C_17_H_18_AuCl_2_N_3_O_4_S_2_, 660.35): Calcd. C 30.92, H 2.74, N 6.36, S 9.71; found C 30.89, H 2.80, N 6.54, S 10.01.

(C_27_H_22_AuCl_2_N_3_O_4_S_2_, 784.49): Calcd. C 41.33, H 2.82, N 5.35, S 8.17; found C 40.99, H 2.74, N 5.45, S 8.31.

### 3.6. In Vitro Cytotoxicity Assay

To assess the cytotoxicity and determine the effective dose, IC_50_, the MTT assay was used. The human cancer cell lines A549 (lung cancer), HeLa (cervical), MDA-MB-231 (breast), and MCF-7 (breast) were cultured in a 96-well plate at 5 × 10^4^ cells/well in quadruplicate in 150 µL DMEM for 24 h. They were treated with cisplatin and complexes (**A**, **1**, **2**, and **3**) at concentrations of 0.3, 1, 3, 10, 30, and 100 µM for 24 h. Then, 15 µL of DMEM containing MTT (3-(4,5-dimethylthiazol-2-yl)-2,5-diphenyltetrazolium bromide) (5 mg/mL) was added to each well and placed in a CO_2_ incubator at 37 °C for 3 h. After incubation, a purple-colored formazan was produced and appeared as dark crystals at the bottom of the wells. The culture medium was discarded from each well and replaced with 100 µL of isopropanol to dissolve the formazan crystals. The absorbance of the 96-well plate was taken at 570 nm with Mithras2LB943 against reagent blank. The IC_50_ value for each sample was calculated using GraphPad 6.0 and Excel 2019.

### 3.7. Apoptosis Analysis for Complex ***2***

To detect the apoptotic effect of complex **2** on MCF-7 cells, we used the (FITC) Annexin V Apoptosis Detection Kit (BioLegend, San Diego, CA, USA). The cells were seeded in 6-well plates, at 5 × 10^4^ cells per well, and treated with different concentrations of complex **2** (0, 1, and 5 µM) for 24 h. After harvesting the cells, cell suspension was stained with FITC Annexin V/7-AAD according to the manufacture’s protocol. Cells treated with a DMSO concentration of less than 0.1% (*v*/*v*) were used as a control. All samples were analyzed using a flow cytometer (Beckman Coulter, FC500, Indianapolis, IN, USA).

### 3.8. DNA Damage of Complex ***2***

Besides apoptosis, the ability to induce DNA damage upon treatment was investigated. MCF-7 cells were seeded in 6-well plates, at 5 × 10^4^ cells per well, and treated with complex **2** at concentrations of 0, 1, and 5 µM for 24 h. Cells treated with a DMSO concentration of less than 0.1% (*v*/*v*) were used as a control. Then, cells were harvested and prepared with the Muse Multi-Color DNA Damage Kit (Merck Millipore, Burlington, MA, USA) according to the manufacturer’s protocol. The percentage of DNA damage was assessed using flow cytometry (Beckman Coulter, FC500, Indianapolis, IN, USA).

### 3.9. ROS Production of Complex ***2***

To estimate the level of ROS, MCF-7 cells were seeded in 6-well plates, at 5 × 10^4^ cells per well, followed by incubation for 24 h at 5% CO_2_, 37 °C, and a 95% humidified environment. Varying concentrations of Complex **2** (1, 5, and 10 µM) were added to each well and incubated for 24 h, with a DMSO concentration of less than 0.1% (*v*/*v*); untreated cells were used as a negative control. After incubation, cells were collected and washed with 1X PBS, following the Muse oxidative stress kit protocol (Merck Millipore, Burlington, MA, USA). Briefly, the washed cells were suspended in 1X assay buffer and then incubated for 30 min at 37 °C after adding the oxidative stress reagent. We estimated the percentage of ROS using flow cytometry (Beckman Coulter, FC500, Indianapolis, IN, USA).

### 3.10. Mitochondrial Membrane Potential of Complex ***2***

MCF-7 cells were seeded in 6-well plates, at 5 × 10^4^ cells per well, followed by incubation for 24 h at 5% CO_2_, 37 °C, and a 95% humidified environment. Varying concentrations of Complex 2 (1, 5, and 10 µM) were added to the each well and incubated for 24 h. Cells treated with a DMSO concentration of less than 0.1% (*v*/*v*) were used as a control. Then, cells were harvested and prepared with the Muse MitoPotential kit (Merck Millipore, Burlington, MA, USA) according to the manufacturer’s protocol. Briefly, 95 µL of MitoPotential and 5 µL of 7-AAD reagents were added to cells. Then, they were incubated for 20 min in a 37 °C CO_2_ incubator. The percentage of depolarized cells was assessed by flow cytometry (Beckman Coulter, FC500, Indianapolis, IN, USA).

### 3.11. Statistical Analysis

All results are reported as mean values ± standard deviation. A one-way ANOVA test was used for all comparison results.

## 4. Conclusions

Three new gold(III) complexes (**1**–**3**) containing 2,2′-bipyridine-3,3′-dicarboxylic acid and dithiocarbamate ligands have been synthesized and spectroscopically characterized. The spectroscopic data supported the coordination of both ligands to the gold(III) ion. In vitro cytotoxicity assay of the complexes demonstrated that they possess very high activity as compared to cisplatin. The results reveal that the dithiocarbamate group plays a significant role in the cytotoxicity of complexes. The apoptosis analyses revealed that the complexes inhibited proliferation, through the induction of apoptosis. The apoptosis induction also triggered DNA damage, oxidative stress (confirmed by accumulated ROS), and depolarization of the mitochondrial membrane, showing the great potential of tested gold(III) complexes (A, **1**–**3**) for anti-cancer activity. The promising results of this study stress the need for further preclinical evaluation of this series of gold compounds to test their clinical potential.

## Figures and Tables

**Table 1:** IR frequencies (cm^−1^) of free ligands and their complexes.

Ligand/Complex	ν (O-H)	ν (C-H)	ν (COO)	ν (N-CS)	ν (C = C)	ν (CSS)
DMDTC	-	-	-	1488	-	926
DEDTC	-	-	-	1445	-	986
DBDTC	-	2923, 2854	1600	1467	1436	985
**A**	3451	2953, 2851	1645	-	-	-
**1**	3448	-	1652	1575	1399	1047, 967
**2**	3440	2975, 2853	1662	1550	1347	1065, 993
**3**	3439	2928, 2854	1716	1549	1435	1067, 904

**Table 2:** ^1^H-NMR chemical shifts for free ligands and their compounds in DMSO-*d_6_*.

Ligand/Complex	H-1	H-2	H-3	H-6	H-8	H-9	Aromatic-Hs
DMDTC	-	-	-	-	3.35 s	-	-
DEDTC	-	-	-	-	3.93 q	1.13 t	-
DBDTC	-	-	-	-	5.31, 4.77 d	-	7.24 m–7.39 m
**A**	8.71d	7.58 t	8.31d	-	-	-	-
**1**	8.67d	7.52 t	8.26 d	-	3.36 s	-	-
**2**	8.69d	7.56 t	8.29 d	-	3.76 q	1.28 t	-
**3**	8.89d	7.77 t	8.78 d	10.13	4.85, 4.76 d	-	7.22 m–7.33 m

s: Singlet; d: Doublet, t: Triplet; q: Quartet; m: Multiplet.

**Table 3:** ^13^C-NMR chemical shifts for free ligands and their complexes in DMSO-*d_6_*.

Ligand/Complex	C1	C2	C3	C4	C5	C6	C = S	C8	C9	Aromatic Cs
DMDTC	-	-	-	-	-	-	208.3	45.7	-	-
DEDTC	-	-	-	-	-	-	206.4	49.5	12.1	-
DBDTC	-	-	-	-	-	-	213.1	56.9	-	127.7–137.2
**A**	157.9	126.8	138.4	123.3	150.3	166.7	-	-	-	-
**1**	158.8	126.6	137.8	122.8	150.6	167	193.9	40.3	-	-
**2**	158.2	126.6	138.1	123	150.3	166.7	193.8	46.5	12	-
**3**	155.9	119.4	142.8	122.8	151.2		193.4	52.1	-	

**Table 4:** IC_50_ values (μM) of cisplatin and gold(III) compounds (**A**, **1**–**3**) against A549, HeLa, MDA-231, and MCF-7 cancer cell lines.

IC50 in μM				
	A549	HeLa	MDA-231	MCF-7
Cisplatin	9.61 (± 0.08)	9.3 (± 0.2)	7.8 (± 0.02)	31.06 (± 0.1)
**A**	7.4 (± 0.07)	7.3 (± 0.03)	5.5 (± 0.02)	15.9 (± 0.021)
**1**	3.02 (± 0.1)	2.8 (± 0.05)	3.3 (± 0.01)	4.08 (± 0.019)
**2**	3.08 (± 0.06)	3.1 (± 0.1)	3.3 (± 0.01)	2.7 (± 0.1)
**3**	3.1 (± 0.5)	3.7 (± 0.07)	4.1 (± 0.03)	3.7 (± 0.019)

## Data Availability

Not applicable.

## Sample Availability

Samples of the compounds are not available from authors.
